# Empathy and Hormonal Changes as Predictors of Sensitive Responsiveness towards Infant Crying: A Study Protocol

**DOI:** 10.3390/ijerph18094815

**Published:** 2021-04-30

**Authors:** Maria Kaźmierczak, Paulina Pawlicka, Paulina Anikiej-Wiczenbach, Ariadna B. Łada-Maśko, Bogumiła Kiełbratowska, Magda Rybicka, Alicja Kotłowska, Marian J. Bakermans-Kranenburg, Marinus H. van IJzendoorn

**Affiliations:** 1 Faculty of Social Sciences, Institute of Psychology, University of Gdansk, Jana Bażyńskiego 4, 80309 Gdańsk, Poland; maria.kazmierczak@ug.edu.pl (M.K.); paulina.anikiej@ug.edu.pl (P.A.-W.); ariadna.lada@ug.edu.pl (A.B.Ł.-M.); 2 Department of Clinical and Experimental Endocrinology, Faculty of Health Sciences, Medical University of Gdańsk, Dębinki 7, 80210 Gdańsk, Poland; bogumila.kielbratowska@gumed.edu.pl; 3 Intercollegiate Faculty of Biotechnology, University of Gdańsk and Medical University of Gdansk, Abrahama 58, 80307 Gdańsk, Poland; magda.rybicka@biotech.ug.edu.pl; 4 Department of Clinical and Experimental Endocrinology, Faculty of Health Sciences, Medical University of Gdansk, Dębinki 7, 80211 Gdańsk, Poland; kotlowska@gumed.edu.pl; 5 Department of Clinical Child and Family Studies, Vrije Universiteit Amsterdam, BT, 1081 Amsterdam, The Netherlands; m.j.bakermans@vu.nl; 6 Department of Psychology, Education and Child Studies, Erasmus University Rotterdam, 3062 Rotterdam, The Netherlands; marinusvanijzendoorn@gmail.com

**Keywords:** sensitive responsiveness, empathy, couple, pregnancy, infant, oxytocin and vasopressin, family

## Abstract

Sensitive responsiveness refers to parents’ ability to recognize and respond to infants’ cues and has been linked to parental empathy. Additionally, oxytocin (OT) and vasopressin (AVP) are hormones important for sensitivity and empathy. The aim of this study is to test the links between dispositional empathy along with changing OT and AVP levels and responsiveness to a life-like doll in couples and to verify whether these factors are predictors of responsiveness to a child’s cues. Exploratory analyses include predictors of sensitive responsiveness: polymorphisms of OXTR, AVPR1a and CD38 genes, personal characteristics and relational factors. The project employs standardized experimental settings that can be used with non-parents and the assessment of parental sensitive responsiveness towards their child. The participants are couples expecting their first child (111) and childless couples (110). The procedure involves caretaking of a life-like doll. Salivary samples and questionnaire data are collected in a planned manner. In the second part, the expectant couples are invited for the assessment of their sensitivity to their own child (Free Play episodes). Parental sensitivity is assessed using the Ainsworth Sensitivity Scale. This paper presents an interdisciplinary research project that reaches beyond the questionnaire measurement, considering many factors influencing the dynamics of adult–infant interaction.

## 1. Background

Sensitive responding refers to parents’ ability to appropriately recognize infants’ behavioral and emotional cues and to respond during interaction in a well-timed, reciprocal and mutually rewarding manner [1]. It is often conceptualized as parental sensitivity or parental sensitive responsiveness to the child’s signals. Therefore, high parental sensitive responsiveness includes prompt and adequate reactions to both verbal and non-verbal stimuli coming from a child [2]. A responsive parent is perceptive during the interaction with a child, which might be reflected by: fulfilling the child’s needs or showing compassion for the fact that these needs cannot be fulfilled; hugging a baby when she or he seems to wish it and putting him or her down when he/she wants to explore the environment; or socially responding to his or her attempts to initiate interaction [3]. Studies on parental responsiveness focus predominantly on mothers instead of fathers. Moreover, studies on parents-to-be are still lacking. Experimental designs using a life-like doll have been proposed to examine the responsiveness of both childless participants and parents [4,5]. Hence, the presented research project focuses on expectant vs. non-expectant couples reacting to a life-like doll and, as a continuation, on the sensitive responsiveness of these new parents towards their children.

Sensitivity to distress is a particularly important dimension of parental responsiveness [6]. One of the indices of such distress is infant crying. It is the most powerful way to provoke a change in an infant’s situation compared to other signaling behaviors, and therefore it is the most apparent early attachment behavior [7]. Such distress vocalizations prompt a parental response in the service of modulating stress and stimulating protection. However, adult reactions to infant crying vary depending on the appraisal of the signal and affective arousal of the receiver. Thus infant–parent interaction is dynamic and is influenced by many contextual factors, as well as the characteristics of not only the infant but also the parent [8,9].

### 1.1. Empathy and Parental Responsiveness

In family psychology, empathy is viewed as an essential component of the childrearing process, although it is rarely studied in the context of personal attributes of mothers and fathers, including those who start their journey to parenthood [10]. Although maternal empathy has been found to be important for developing a bond with a child and responding to the baby’s needs, a less studied question is the role of partners’ empathy in their relationship with the mother, especially in the context of parental roles [11].

Empathy is a multidimensional construct, which includes the tendency to experience empathic emotions (personal distress—emotional contagion (self-focused); empathic concern for others—sympathy (other-oriented) as well as taking the perspective of others (other-oriented cognitive empathy)) [12,13]. Other-focused, empathic individuals display emotional regulation while dealing with stressful situations, and therefore empathy facilitates the adaptation to life transitions, including the transition to parenthood [14]. In contrast, personal distress is a predictor of worse communication in couples after the transition to parenthood [15,16]. Thus, individual differences in empathy are best revealed in situations of strong, negative emotions concerning close relationships [12,13].

The infant cry is a stimulus coming from a child that evokes strong emotional reactions, activating both appetitive and aversive reactions [17] in parents, and is difficult to ignore. Parental empathy facilitates the sensitive responsiveness to child’s needs [10,18] as it involves regulation of emotions accompanied by self–other distinction [12,13]. Highly dispositionally empathic parents display tendencies to focus on the child’s needs when he/she cries [19]. On the contrary, low empathy favors self-focus instead of interest in the child’s well-being [20]. It is also possible that not every dimension of empathy is beneficial for responsiveness to infant crying. Associations of personal distress with lower emotional regulation abilities and with higher intensity of experienced negative emotions may lead to concentration on one’s own experiences [21,22].

In the HEART research project (HORMONALLY mediated EMPATHY role for AFFILIATIVE RESPONSE towards infant TEARS), we test which empathic dimensions are predictors of sensitivity in parental roles, operationalized as sensitive responsiveness towards infant crying in couples expecting their first child.

### 1.2. Hormonal Levels as Mediators between Empathy and Responsiveness to Child Cues

We expand earlier research on empathy by including measures of oxytocin (OT) and vasopressin (AVP) as hormones important in close relationships, and also in the context of parental roles [23,24,25]. Both hormones have been hypothesized to be linked to empathy, trust in relationships, forming social bonds and attachment processes or parental sensitivity (for a review, see [26]), but most of these studies seem to have focused on OT. Not only does OT facilitate social approach behavior, but it also attenuates endocrine and autonomic responses to stress [27]. Higher levels of OT are viewed as an important factor for mother and infant adaptation, a predictor of empathy and development of the bond with a newborn child [28,29] and sensitivity to infants’ cues. However, for fathers, parenting research has also pointed to a role for AVP levels [23]. Hence, one of the central questions in the project is whether dispositional empathy in couples facilitates sensitive responsiveness towards child cues and signals of distress in the caretaking situation, and whether this association is (partially) mediated by OT and AVP levels.

### 1.3. Additional Biological Factors in the HEART Project

Since OT and AVP levels are central to our research questions, we also include single-nucleotide polymorphisms (SNPs) within human OXT–AVP pathway genes (OXT, AVP, AVP receptor 1a (AVPR1a), OXT receptor (OXTR) and ADP-ribosyl cyclase (CD38)) as distant predictors of hormonal levels and their changes, empathy and responsiveness towards child signals. Recent studies on CD38 revealed that this transmembrane protein elaborates on the release of the OT (Jin et al., 2007). Moreover some transcription factors may be also involved in OT expression in the brain (e.g., SIM1 in the hypothalamic paraventricular nucleus (PVN)) and therefore may be associated with social functioning (Hovey, et al. 2017). The selected SNPs have been previously reported to have an impact on social behavior [30] and other-oriented emotional and cognitive empathy [31,32,33,34,35]. Nevertheless, research in this field should be considered at the exploratory stage as the meta-analysis regarding selected SNPs of OXTR does not support the above-mentioned effects [36]. Thus, a novel perspective is the inclusion of more SNPs and analyzing empathy in the particular context—here, in couples [37,38,39]—and parental sensitive responsiveness [40,41,42,43].

### 1.4. Additional Psychological Factors in the HEART Project

In the presented project, psychological predictors of sensitive responding towards a child’s cues refer to individual characteristics (attachment styles, temperament), and the assessment of important relationships (retrospective assessment of parental attitudes and satisfaction with an intimate relationship in adulthood) [44]. In this respect, personal characteristics should be taken into consideration, such as attachment style [45] or factors such as temperament or sensory sensitivity [3], that might make individuals more or less susceptible to contextual factors and their influence on responsiveness to infant crying [4]. The perceived quality of close relationships (such as satisfaction with intimate relationship or partners’ empathy towards each other) might be a major contextual factor (positive or negative) for sensitive responsiveness towards infant crying, including reactions towards a life-like doll [11,46]. In earlier studies, the role of the parental physical response to infant crying sounds was analyzed [47]. It was concluded that experiencing adversity in childhood might dampen the positive effects of experimentally increased levels of OT [47].

## 2. Research Question

Referring to the attachment framework, the HEART project focuses on sensitive responsiveness, displayed by the couple in parental roles (when they imagine themselves as parents, also during the transition to parenthood). The main aim of the proposed study is to test the link between dispositional empathy and sensitive responsiveness to infant crying—a signal that evokes parental physiological, emotional, cognitive and behavioral responses. Going beyond previous research on mothers [19], responses to infant crying in couples are examined and mediating hormonal levels are assessed.

The HEART project methodologically reaches beyond self-report measurement, and combines personality as well as genetic and endocrine measures, stressing the importance of individual differences for the functioning of couples in the context of parental roles. There is a need for studying empathy and parental sensitivity in couples. Personality, hormonal and couple relationship factors are examined as predictors of responsiveness towards infant crying. To test the individual differences in parental sensitive responsiveness, we implement an experimental design with the use of a life-like doll to be used with expectant and non-expectant couples (a standardized experimental setting for the assessment of sensitive parenting that can also be used with non-parents) and the subsequent assessment of sensitive responding towards own child for the couples that participated in the study during pregnancy. With this design, we are able to control many factors influencing the dynamics of adult–infant interaction. Affective states experienced by participants evoked by a crying signal will also be examined in the context of personality, hormonal and couple predictors. To the best of our knowledge, the project also constitutes the first attempt to include retrospectively assessed parental attitudes in the family of origin along with genetic factors and levels of OT and AVP in their prediction of sensitive responsiveness towards infant crying.

### Aims and Hypotheses

**Figure 1:**
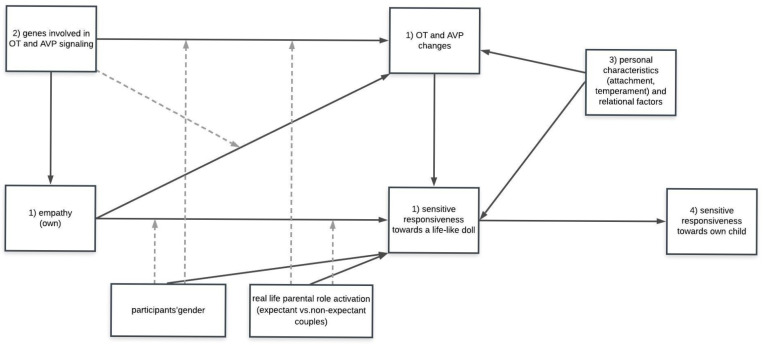
Overview of the most important predictors and outcomes, moderators and mediators. The numbers of the variables refer to the different aims of the couple and the family studies.

1. The primary aim of the HEART study (see Figure 1 for an overview of the different aims) is to test the links between dispositional empathy, OT and AVP levels and sensitive responsiveness to infant crying, assessed in couples, using the infant simulator. We measure responses to infant crying in two ways: subjectively, using self-reported emotional reactions towards infant crying, and objectively, using observation of participants’ behaviors. We expect that higher empathy (higher EC, PT and PD) and higher oxytocin (OT) and vasopressin (AVP) basal levels and reactivity will predict higher individual and couple’s sensitive responsiveness towards infant crying. Moreover, due to the fact that both empathy and parental responsiveness might be gender- and context-specific, we aim to analyze whether gender and real-life parental role activation (expectant vs. non-expectant couples) predict the levels of individual and couples’ sensitive responsiveness towards infant crying and whether this association is (partially) mediated by changing OT and AVP levels during the standardized procedure of taking care of the life-like doll. We also aim to test whether gender and parental role activation moderate the associations between dispositional empathy and sensitive responsiveness towards a life-like doll and between genetic polymorphisms and OT and AVP changes.
2. An exploratory second aim was as follows. OT and AVP might also be linked to genetic polymorphisms in the genes involved in OT and AVP signaling. Thus, our secondary question is whether polymorphisms of OXTR, AVPR1a and CD38 genes predict empathy as well as changes in OT and AVP levels across the experimental setting, and thus predict sensitive responsiveness towards infant crying. Interactions between genetic polymorphisms, hormonal levels and empathy will be also analyzed.
3. At the tertiary exploratory level, we explore whether other personal characteristics (attachment style, temperament) and relational factors (perception of partner, relationship with partner and retrospectively assessed parental attitudes in the family of origin) predict sensitive responsiveness towards infant crying, independently and through hormonal mediation.
4. Although the main goal of the current study is to examine the predictive effects of empathy and hormonal factors on sensitive responsiveness to infant crying in a standardized setting before the birth of the couples’ own child, we also aim to test whether these factors are predictors of responsiveness to their own child’s cues. Therefore, we plan to continue the research in the sample of expectant couples to examine sensitive responsiveness in mothers and fathers towards their own children aged 6–12 months.

## 3. Methods/Design

The HEART project employs standardized experimental settings for the assessment of parental sensitive responsiveness that can be used with non-parents and—in a later stage of the project—the assessment of parental sensitive responsiveness towards their own child. The participants are couples living in the northern part of Poland. Only couples with a relationship lasting at least 2 years and with previous cohabitation can participate. Due to the need for homogeneity of the sample, only heterosexual couples participate in the study.

The procedure involves caretaking of a life-like doll, which cries as programmed during the interaction with a caregiver. This standardized procedure creates a unique opportunity to observe responsiveness to infant distress signals, which is difficult to obtain in observations of free play with a child [46]. Therefore, as the project’s continuation, the expectant couples are invited for an assessment of their parental sensitive responsiveness towards their own child’s cues 6–12 months after the child’s birth (objectively, during a free play procedure, and subjectively, with the Parental Responsiveness Scale (SRR; [48,49])).

All parts of the project are conducted in the laboratory with a two-way mirror and cameras situated in the Institute of Psychology at the University of Gdansk furnished as an infant room. Another laboratory room for gathering questionnaire data and storage of the salivary samples is arranged in the Institute of Psychology. Each session takes place during the same time of the day in order to avoid the influence of diurnal variations concerning hormone secretion [50].

### 3.1. Participants

In total, 597 couples received an invitation (oral or written) to participate in the study. A third of the couples (34.51%) did not respond to the invitation, 148 (24.79%) did not meet the inclusion criteria, 22 (3.69%) of the couples refused to participate, and one couple resigned during the examination. A final sample of 221 couples (37.02%; including 111 couples expecting their first child and 110 couples in the control group) participated in the study. The background information collected through the registration form was obtained from 171 couples not participating in the study. Their characteristics compared with the study group are listed in Table 1. Any significant differences between participating and non-participating couples were negligible in size. Moreover, Table 2 presents pregnancy characteristics of participating and non-participating couples. The non-participating ones tended to fill out the registration form earlier during pregnancy than participating couples (medium effect size). Differences were measured using *t*-test for independent samples and the effect size was measured with Cohen’s d.

### 3.2. Recruitment

**Figure 2:**
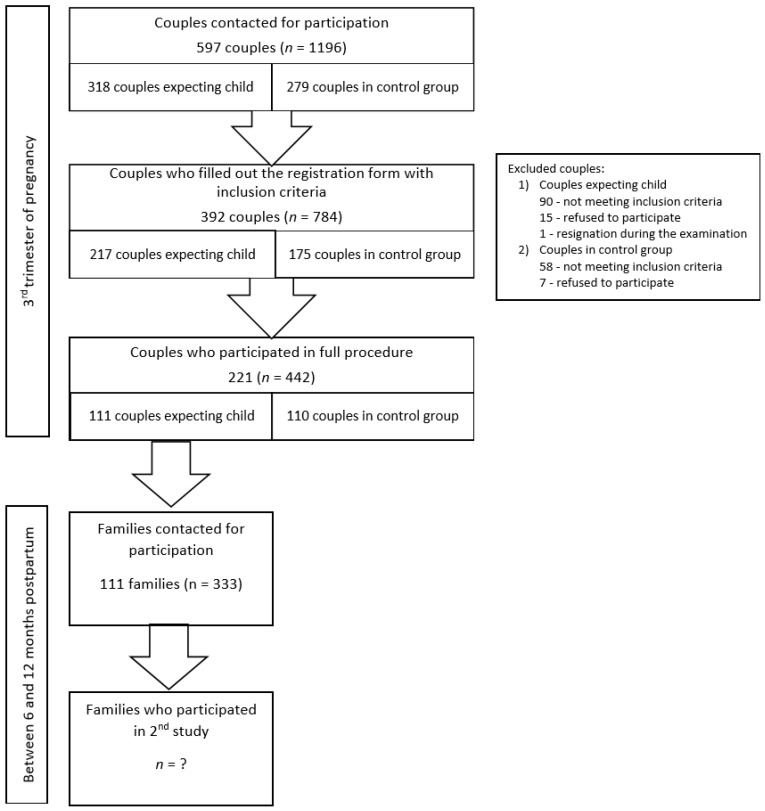
Flow chart of the number of participants in the first (expectant and non-expectant couples) and the second (between 6 and 12 months postpartum) session.

Couples expecting their first child were contacted via antenatal schools in the Pomeranian area and social media. Non-expectant couples were recruited via announcements at the University of Gdansk and social media. In order to be included, all participants had to be in the age range 22–35 years. Only couples with a relationship lasting at least 2 years and with previous cohabitation could participate. Exclusion criteria for both partners were tobacco consumption, drug use, pharmacological treatment such as glucocorticosteroids or selective serotonin reuptake inhibitors, alcohol and psychoactive substance addiction. All participants had to declare general good health. The two groups (expectant vs. non-expectant) should not have significantly differed from each other on sociodemographic characteristics. Additionally, the inclusion criteria for the postpartum session were participation of the whole family (both parents and their first child), age of the infant between 6 and 12 months and absence of developmental disorder diagnosis. The recruitment process is presented in Figure 2.

### 3.3. Procedure

The participants were asked not to consume spicy and salty foods, alcohol, coffee and tea as well as foods with high sodium levels in the 24 h prior to the session and sampling. Upon arrival at the lab, and 30 min prior to the observation of the first person in the couple, two salivary samples were collected from each partner to measure basal salivary OT and AVP, and to gather genetic material for genotyping of polymorphisms in OXTR, AVPR1a and CD38 genes. Next, each partner started to fill in the psychological questionnaires on the computer, and after filling in the first questionnaire, one of the partners, in random order, was assigned to participate in a 10 min episode with a life-like doll for the assessment of the participant’s sensitive responsiveness, while the other continued to fill in the questionnaires. Next, the same 10 min episode with a subsequent partner followed. Then, the third 10 min episode with the couple (couple’s sensitivity) was conducted. Each session was followed by filling in the PANAS-X in Polish [51] and some questions on the reality of the situation in the Experiences with the Infant Simulator [46]. Additionally, participants completed the My Emotions Scale, measuring their emotional reactions to infant crying [52]—own (first and second episode) and partner’s (third episode). Sensitivity was assessed using the Ainsworth Sensitivity Scale (individual sensitivity towards a life-like doll—the first and the second episode; couple’s sensitivity towards a life-like doll—the third episode). After the third episode, participants also assessed their partners’ reactions on three Likert’s response scales, for being supportive, being engaged in communication and depreciation. Then, 15 min after each session, a second salivary sample was gathered from each partner to measure the level of salivary OT and AVP.

#### 3.3.1. Stimulus: The Infant Simulator

The infant simulator (RealCare Baby 3; Realityworks, USA) is a doll resembling a real infant in physical appearance, size and weight (2.95 kg). It makes breathing, burping, giggling, suckling and crying sounds and needs to be cared for like a real infant. The doll used in the project was a Caucasian female or male infant (for expectant couples, the gender of the simulator complied with the gender of their own baby; for non-expectant couples, the simulator was randomly assigned) dressed in a long-sleeved sleeper and a blanket, thus creating the impression of holding a real baby. The simulator was equipped with software enabling pre-programming of its behavior using schedules based on real infants. Additionally, thanks to electronic wristbands, the noises of the simulator could depend on the care provided (option responsive to care) or could be pre-set independently of the care provided (pre-set option).

Since our study was observational and the infant simulator served as a stimulus for assessment of the sensitivity, a pre-set option (according to Voorthuis et al., 2013, p. 607) was chosen in order to provide equal session conditions for all participants. The infant was crying with a varying frequency. The cry began in the third minute with around 30 s of mild fussing and then became full-blown crying for 4 min. The infant simulator stopped crying in the last minute of the session. All participants received the same instruction: “Please, take care of this infant until I come back. The whole room is at your disposal”. The doll was given to men to encourage their engagement in the task (stereotypically female). The procedure took place in a laboratory furnished like an infant room equipped with a crib, a car seat/carrier, a sofa, an armchair, a baby changing table and numerous accessories placed all over the room: a bottle, a blanket, clothes, diapers, board books and toys (rattles, squeeze and soft toys, teething toys, textured balls). Thus, participants had the opportunity to behave as in a real caregiving situation.

#### 3.3.2. Postpartum Session

The participants from the expectant group were invited to the subsequent session when their child was between 6 and 12 months old. Every family came to the same room arranged to allow playing with the child. One of the parents was asked to play with his/her child as usual for a few minutes (free play procedure). At the same time, the second parent filled in the questionnaires. Then, they swapped places. Sensitivity during the episode with the child was rated using the observational scale (the Ainsworth Sensitivity Scale) and self-reportedly with the Parental Responsiveness Scale (SRR; [48,49]). Moreover, Cooperation vs. Interference with Baby’s Ongoing Behavior were also assessed using the Ainsworth Scale. Additionally, participants filled in the My Emotions Scale, measuring emotional reactions to their own baby’s cry [52]. The demographic data were also collected (e.g., education level, employment and level of satisfaction with economic status, child’s gender, data about labor). The postpartum session took less than one hour.

## 4. Measures

### 4.1. Empathy

Dispositional empathy was assessed using the Empathic Sensitiveness Scale (SWE; [53]). SWE is a 28-item measure with a 5-point scale, which contains three subscales measuring components of empathy: Empathic Concern (EC; 11 items), Personal Distress (PD; 8 items) and Perspective Taking (PT; 9 items) [53]. The reliability of this questionnaire in the current study was α=0.82, and for the subscales, it was α=0.79 for EC, α=0.77 for PD and α=0.73 for PT.

### 4.2. Sensitive Responsiveness

Self-report. For the subjective assessment of sensitive responsiveness, the Polish adaptation of the My Emotions Scale [54], the Infant Crying Questionnaire (SER-PD; [52]), was used. The scale measures parent-oriented (Anxiety, Frustration and Amusement) and infant-oriented (Sympathy and Empathy) emotional reactions to a child’s cry. SER-PD consists of 20 items, with a 5-point Likert response scale, where each subscale consists of 4 items. The scale was modified for use in our study. Participants evaluated the frequency of their particular emotional reactions to the child simulator crying during the experiment as their imagined own baby. The Cronbach’s α for the SER-PD questionnaire in the present study was 0.72. The reliability for the parent-oriented subscales was: α=0.77 for Anxiety, α=0.71 for Frustration and α=0.81 for Amusement, and for the infant-oriented subscales, it was α=0.80 for Sympathy and α=0.74 for Empathy.

The Parental Responsiveness Scale (SRR; [48,49]) is used for subjective assessment of responsive reactions during a parent’s interactions with their own child. This questionnaire consists of 15 items, with a 7-point response scale (where 1 means “I strongly disagree” and 7 means “I strongly agree”). The reliability of the SRR scale will be calculated after the second part of the study is carried out.

Observation. The Ainsworth Sensitivity Scale [55] was used for observing sensitive responsiveness to the infant simulator’s crying and the participants’ own infants’ cues. In order to assess sensitivity when taking care of the infant simulator, the assessment with use of the Ainsworth Sensitivity Scale was modified [46]. Such aspects as the intensity of the signals being noted, interpretation of the signals, appropriateness (trying various soothing strategies, e.g., feeding, changing a diaper, rocking, carrying) and promptness of responding were taken into account. The assessment of sensitivity towards participants’ own infant child consisted of two scales, the Sensitivity Scale and the Cooperation Scale [55], measuring the cooperation vs. interference with the child’s ongoing behavior.

### 4.3. OT and AVP

Saliva samples for the evaluation of OT and AVP levels were collected from participants by applying salivettes and subjected to the process of lyophilization in order to increase the stability of hormones and ensure that the concentration of OT and AVP exceeded the limits of detection and quantification of the radioimmunoassay (RIA) test [56,57,58]. The liophilizates were stored at −40 ${}^{\circ }$C until the moment of analysis.

### 4.4. Genotyping

On the basis of a literature search, we selected 10 single-nucleotide polymorphisms (SNPs) in OXTR and CD38, and 2 simple sequence repeats (SSRs) in AVPR1a. Genotyping of 10 SNPs was performed with the use of the MassARRAY^®^ Analyzer (Agena Bioscience, San Diego, CA, USA), which utilizes matrix-assisted laser desorption/ionization time-of-flight mass spectrometry for qualitative nucleic acid analysis.

### 4.5. Personal Characteristics

Attachment style. We used a short version of the Experience in Close Relationships—Revised (Polish translation, DBZ-R; [59]). The scale assesses adult attachment in close relationships based on avoidance and anxiety indicators. The short version consists of 16 items with a 7-point Likert response scale.The Cronbach’s α for DBZ-R in the present study was 0.70, with α=0.87 for the Anxiety subscale and α=0.87 for Avoidance.

Temperament. The short version of the Formal Characteristics of Behavior—Temperament Inventory (FCZ-KT; [60]) was used to assess temperamental characteristics, according to the Regulatory Theory of Temperament, and refers to the formal aspect of behavior. It consists of 42 items with a YES/NO answer format and has the following subscales: Briskness, Perseverance, Sensory Sensitivity, Emotional Reactivity, Endurance and Activity. The FCZ-KT reliability in the present study was α=0.48.

### 4.6. Relationship Characteristics

Perceived partner empathy. The perception of a partner’s empathy was assessed with the Perceived Partner’s Empathy Scale (PPE; the description of the scale, [61]). The unidimensional measure consists of 20 items, which describe partner’s empathy (empathic concern and perspective taking; other-oriented components, beneficial for relationships’ quality) in their relationship. On the 5-point rating scale, partners assess each other’s reactions [62]. The reliability for this scale in the present study was α=0.87.

Relationship satisfaction. We used a scale that comes from the RELATionship Evaluation Questionnaire [63,64] and is the part of the assessment of romantic relationships in various moments of life by Hawkins [65], Polish adaptation by Rostowska and Kaźmierczak (unpublished scale; previously used, e.g., [62]). This 7-item scale measures relationship satisfaction in various aspects (e.g., physical intimacy, time together) with a 5-point response scale. The present satisfaction with relationship in comparison with expectations was also measured in the study using the Cantril’s Ladder [66] in a modified form to test relationship general satisfaction. The Cronbach’s α for RELAT in the present study was 0.80.

### 4.7. Retrospective Assessment of Parental Attitudes in the Family of Origin

The participants retrospectively assessed the behaviors of each of their parents (separately mother and father) using the Family of Origin (RP) [67], which refers to democratic, autocratic, liberal loving and liberal unloving parenting styles according to Field [68]. Each of the two questionnaires consists of 34 items with a 5-point Likert scale. The reliability for the RP questionnaire in the present study was α=0.44 for mothers, and α=0.41 for fathers. Moreover, the Cronbach’s α for subscales was 0.89 for democratic parenting style of mothers, 0.85 for autocratic, 0.52 for liberal loving and 0.83 for liberal unloving, and for fathers, the Cronbach’s α was 0.91 for democratic parenting style, 0.86 for autocratic, 0.66 for liberal loving and 0.87 for liberal unloving.

## 5. Sample Size and Power

For our primary aim, testing the direct and indirect (through oxytocin and vasopressin) effect of dispositional empathy on responsiveness to infant crying, with α=0.05, effect size of f=0.35 and sample size of 221 couples, the power is >90% (multiple linear regression: fixed model, $R^{2}$ deviation from zero, G*Power 3.1.9.2). For our secondary aim, testing whether polymorphisms of OXTR, AVPR1a and CD38 genes predict empathy and interact and predict OT and AVP reactivity and sensitive responsiveness towards infant crying, and tertiary (whether personal characteristics and relational factors predict sensitive responsiveness towards infant crying, independently and through hormonal mediation) aims, testing mediation effects, the power is >80%. For the fourth aim, based on a total sample size of 111 couples expecting a child, due to the difficulty of situation, which is later adaptation to parental roles or possible life changes after the child is born, we have estimated that potential loss to follow-up may be in the order of 30–40%, which leaves 66 to 77 families (couples and a child) with follow-up data. This would allow between 70% and 80% power to detect standardized effect sizes of 0.15 with α=0.05.

## 6. Statistical Analyses

The data analysis will be carried out using R [69] with the lavaan [70] and psych [71] packages, after the study and data collection have been finished. We will apply range checks for data values in order to check measurement models (e.g., using confirmatory factor analysis) and the reliability of the entered data, and possible outliers will also be identified and then winsorized.The exploratory analyses will be carried out to test whether sociodemographic factors such as gender, age, level of education, marital status and work situation impact the responsiveness towards infant crying, parental sensitivity and associations between variables in the research model. If necessary, data transformation will also be carried out to bring the distribution of variables closer to normal distributions. For the primary aim, we propose a path analytic framework for integrating mediation and moderation, as well as structural equation modeling (for latent variables). The model will test whether oxytocin (OT) and vasopressin (AVP) levels mediate the effect of the dispositional empathy on the sensitive responsiveness to infant crying. Our secondary aim is to examine whether polymorphisms of OXTR, AVPR1a and CD38 genes predict empathy and interact and predict changes in OT and AVP levels and thus responsiveness towards infant crying, so again we propose to use mediation (with empathy and/or hormonal OT and AVP levels as mediators) analysis and also moderated (polymorphisms of OXTR, AVPR1a and CD38 genes as moderators) mediation analysis to test the above hypothesis. The third and fourth aims of our study also require the use of path analysis and consideration of possible mediators and moderators. The significance of obtained mediational effects will be checked using bootstrapping procedures. Unstandardized indirect effects will be computed for each of at least 1000 bootstrapped samples, and the 95% confidence interval will be computed by determining the indirect effects at the 2.5th and 97.5th percentiles [72,73,74]. Furthermore, analysis of variance (ANOVA) will be carried out to analyze the differences among group means in a sample (e.g., expecting and non-expecting couples) in terms of studied variables. Additionally, the actor–partner interdependence model (APIM) will be used for measuring effects in interpersonal relationships in couples on their sensitive responsiveness to the infant cry. The explorative analyses will be carried out to test whether sociodemographic factors impact the responsiveness towards infant crying, parental sensitivity and associations between variables in the research model.

## 7. Data Management and Ethics

The project collects novel data from 442 Polish women and men using a secure web-based environment at the University of Gdansk, Poland. Access to data during the research period is restricted to project members (including principal investigators and research assistants) given access to the data. All members of the research team have signed a confidentiality agreement.

Personal information is processed in accordance with the Polish Personal Information Protection Act based on European legislation. All the data are coded and a separate participant identification code list used to link the data and biological specimens is stored until the end of the research project in a separate location in order to secure the personal information. Results will be mainly published in peer-reviewed international journals in accordance with the project description and agreements within the consortium. There will be no personal identification of participants in scientific communications.

Written informed consent regarding all collected data (psychological information, physiological material samples, audiovisual material) is obtained from the participants. They are also reminded that participating in the study is voluntary, that they can withdraw from it at any time without consequences and that their data are stored anonymously and securely. All consent forms and related documentation given to the participants were approved by the Ethics Committees and can be requested from the authors. The research protocol received ethical approval by the Independent Bioethics Committee for Scientific Research at Medical University of Gdańsk, Poland (permission #NKBBN/154/2017) and the Ethics Committee at the Institute of Psychology, University of Gdańsk, Poland (permission #4/2016) and (permission#6/2018), and is in accordance with the 1964 Helsinki Declaration and its later amendments or comparable ethical standards. The study is registered in the Open Science Framework (https://osf.io/z5682/, accessed on 22 January 2020).

## 8. Discussion

This study protocol presents an interdisciplinary research project that methodologically goes beyond the questionnaire measurement, and combines personality and physiological measures. In HEART, we examine the influence of individual differences on couple functioning in the context of parenting. Studies on parenting urgently need to include men, who rarely take part in research on empathy and parental sensitivity/responsiveness.

To test individual differences in parental sensitive responsiveness and to assess the impact of dispositional empathy on parental sensitivity—direct and indirect, through hormonal mediators—we implement an experimental design with the use of a life-like doll (a standardized experimental setting for the assessment of sensitive parenting that can be used with non-parents), and focus on the crying signal. Using this design, we are able to control many factors influencing the dynamics of adult–infant interaction. Affective states experienced by participants evoked by a crying signal are also examined in the context of biological and psychological predictors. Additionally, we examine parental sensitive responsiveness of couples towards their own child’s cues in follow-up assessments, which uniquely enables exploration of the predictive validity of the experimental design with a life-like doll.

Therefore, HEART explores the issues essential for the quality of family life and children’s development. We seek answers to questions about the bases or roots of the role of empathy in displaying sensitivity towards children’s needs and thus in creating adult–child bonds. Sensitive parental behaviors in response to babies’ signals have an impact on the formation of proper bonds, the development of the child’s social cognition and emotional regulation [2,75]. The presented studies on empathy underpin its beneficial role for parenthood as it facilitates a focus on children’s needs, also when they are in distress. An infant’s cry is a strong cue that prompts parental response. Both empathy and sensitive responsiveness have been linked to hormonal changes, and oxytocin has been deemed crucial for close relationships including parental empathic responses. Thus, the above described research indicates that empathy and hormonal levels might predict higher sensitive responsiveness towards infant crying. In times of heightened independence, with increased social isolation in societies, and increasing frequency of divorce and pathology in families, a deepened analysis of ‘a social glue’—that is, empathy—for sensitive responsiveness to children’s signals is required. The description of the project’s aims and procedure might be of interest also to practitioners. Since the diagnosis of a child requires taking into account a wide range of factors and requires specific procedures and the skills of psychologists, our project follows a new trend of implementing multimodal simulators in psychology. Additionally, we propose that the sensitive responsiveness towards children is one of the key constructs for practitioners that sheds light on a method to assess parenting quality and its predictors. Thus, the presented standardized observational procedure will improve the diagnostic process of families with young children.

## 9. Strengths and Limitations

The study has some limitations. The study design might facilitate participation of couples who are well educated or highly involved in their future parenthood (e.g., recruiting participants at antenatal classes or universities), which might limit the possibilities for generalization. In the second part of the study, it is not possible to invite a control group, due to the specificity of the research measures including, the Ainsworth Sensitivity Scale and Parental Responsiveness Scale. Additionally, hormonal measurement requirements limit the heterogeneity of the sample as only healthy couples who were not taking particular medications and psychoactive substances participated in the studies. The experimental procedure with a life-like doll yields some risk that particular participants will not consider this controlled situation realistic. However, exploration of individual differences in this standardized setting will give the opportunity to examine characteristics that are associated with lack of engagement and low response to the experimental cues. The experimental situation as well as the subsequent observation of the free play with the participants’ own children might not be long enough to examine individual differences in parental sensitivity. However, similar designs have been successfully implemented before [46].

The HEART project enables testing of the predicted positive impact of dispositional empathy and hormonal factors—OT and AVP—on parental sensitivity in a controlled experimental design (expectant vs. non-expectant couples participating in the same experimental procedure twice, individually and together), which constitutes a major strength. The large sample, including both expecting and non-expecting couples, gives the opportunity to test the impact of parental role activation on responsiveness towards infant crying. In a standardized setting, use of a life-like doll enables manipulation of a child’s cues that might elicit sensitive responsiveness. The research design uniquely combines psychological (correlational and observational) measures with biological assessments. Measures have been translated into Polish to examine sensitive responding subjectively, through self-reported emotional reactions towards infant crying, and objectively, through observation of participants’ behaviors. Finally, the postpartum session is designed to examine sensitive responsiveness towards participants’ own children’s cues (objectively and subjectively with a new measure) in the group of formerly expectant couples, which provides the possibility to test the predictive validity of the study’s experimental procedure. Therefore, HEART results might be clinically used to design interventions and programs for parents (also among childless women and men) to support and develop their skills in sensitive responding towards child cues.

## Figures and Tables

**Table 1:** Demographic characteristics of all couples who filled out the registration form with inclusion criteria.

	Participating Couples (*n* = 221)	Non-Participating Couples (*n* = 171)		Effect Size
Age at recruitment M (SD)	26.66(3.24)	28.01(4.02)	p=0.001	d=0.37
Duration of relationship (years)	5.76(3.04)	5.87(3.73)	p=0.022	d=0.03

Note. *n* refers to couples.

**Table 2:** Characteristics regarding pregnancy and children of participating and non-participating expectant couples.

	Participating Couples Expecting a Child (*n* = 111)	Non-Participating Couples Expecting a Child (*n* = 105)		Effect Size
Pregnancy				
*single*	111	103		
*twin*	0	2		
Week of pregnancy				
at the time of recruitment	31.32(4.58)	30.26(6.42)	p=0.005	d=0.19
Sex of an expected child				
*girl*	65(58.56%)	42(40%)		
*boy*	41(36.94%)	51(48.6%)		
*unknown*	5(4.50%)	12(11.4%)		
Prenatal depression	15.07(3.15)	14.70(3.49)	p=0.020	d=0.11

Note: *n* refers to couples.

## Data Availability

The data presented in this study are openly available in Open Science Framework Storage Repository at https://osf.io/xebhg/ (accessed on 14 April 2021).

## Trial Registration

osf.io/Identifier: osf.io/z5682 (accessed on 22 January 2020).

## Sample Availability

The data that support the findings of this study are not publicly available due to ongoing analyses.
